# Periodontal Management of Sturge-Weber Syndrome

**DOI:** 10.1155/2013/517145

**Published:** 2013-05-20

**Authors:** Butchibabu Kalakonda, Koppolu Pradeep, Ashank Mishra, Krishnanjaneya Reddy, Tupili Muralikrishna, Vijaya Lakshmi, Radhika Challa

**Affiliations:** Department of Periodontics, Sri Sai College of Dental Surgery, Vikarabad 501101, India

## Abstract

Sturge-Weber syndrome (SWS) is a sporadic disorder and is frequent among the neurocutaneous syndromes specifically with vascular predominance. This syndrome consists of constellation of clinical features like facial nevus, seizures, hemiparesis, intracranial calcifications, and mental retardation. It is characterized by focal port-wine stain, ocular abnormalities (glaucoma), and choroidal hemangioma and leptomeningeal angioma most often involving occipital and parietal lobes. The present paper reports three cases of SWS with oral manifestations and periodontal management, which included thorough scaling and root planing followed by gingivectomy with scalpel and laser in cases 1 and 3 consecutively to treat the gingival enlargement. However, the treatment in case 2 was deferred as the patient was not a candidate for periodontal surgery.

## 1. Introduction

Sturge-Weber syndrome (SWS) is a sporadic disorder that occurs with frequency of 1 : 50,000 births [1]. Inspite of being rare, it is the most frequent disease among the neurocutaneous syndromes specifically with vascular predominance [2]. It is sporadic neurocutaneous disease characterized by focal port-wine stain, ocular abnormalities (glaucoma), and choroidal hemangioma and leptomeningeal angioma most often involving occipital and parietal lobes [3]. This syndrome consists of constellation of clinical features like facial nevus, seizures, hemiparesis, intracranial calcifications, and mental retardation [4].

Encephalofacial angiomatosis and encephalotrigeminal angiomatosis are used as synonyms of this syndrome as angiomas involve the leptomeninges and skin of face typically in ophthalmic and maxillary distributions of trigeminal nerve. SWS was initially described by Schirmer in 1860 and named by William Allen Sturge (1879) and Fredrick Parkes Weber (1929) [5].

Sturge associated the dermatological and ophthalmic changes with diseases of neurological manifestations, and Weber demonstrated gyriform intracranial calcifications and termed them “tramline,” “tram-trac,” or “trolley track” calcifications.

The present paper reports three cases of Sturge-Weber syndrome with oral manifestations and management.

## 2. Case Presentation

*Case 1*. A 23-year-old male patient came to the Department of Periodontics, Sri Sai College of Dental Surgery at Vikarabad, with a chief complaint of swelling in upper front region for 4 years which was gradually increasing in size with associated bleeding gums while eating or sometimes spontaneously. He gave no history of convulsions or glaucoma and no relevant family history.

Extraoral examination revealed the port-wine stain or nevus flammeus on the left side of the face.

**Figure 1:**
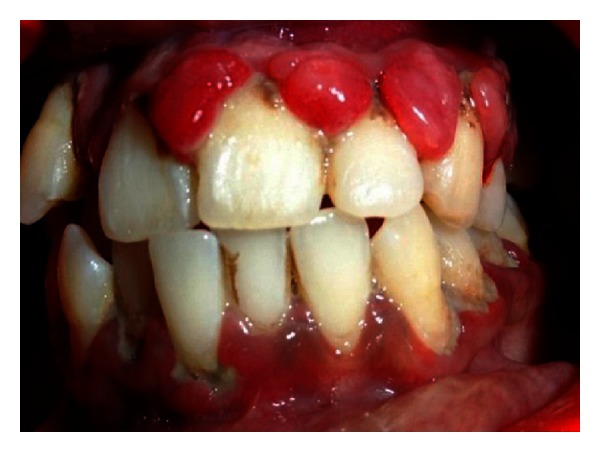
Intraoral view showing unilateral labial overgrowth.

**Figure 2:**
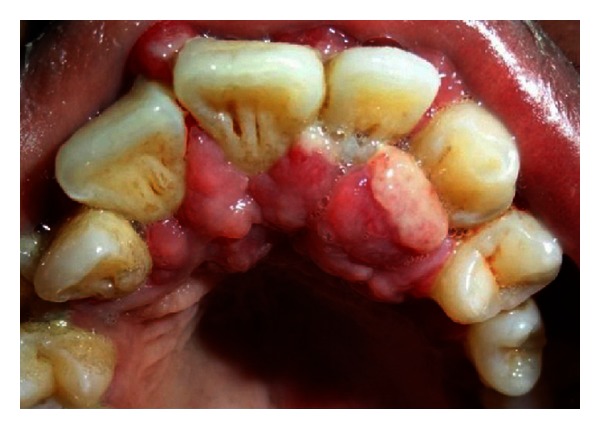
Intraoral view showing unilateral palatal overgrowth.

The patient's mother had informed us that this stain was present on the face since his birth. Intraoral examination (Figures 1 and 2) reveals severe gingival overgrowth which was discrete, pedunculated, reddish-pink in relation to 11, 21, 22, 23, 24, and 25 involving marginal and attached gingiva and was suggestive of an inflammatory gingival overgrowth. The overgrowth extended palatally too in relation to 11, 21, 22, 23, and 24. Loss of Knife edge contour and bulbous papillae are seen in relation to 11 to 24 both labially and palatally. There was slight blanching of the gingival enlargement on pressure raising concerns that it might be an angiomatous (vascular) nature of gingival enlargement. Radiographic and blood investigations were carried out which revealed no abnormality. Histopathological examination was suggestive of inflammatory fibroepithelial hyperplasia with vascular proliferation. Detailed clinical examination, history, and investigation confirmed the diagnosis of Sturge-Weber syndrome.

*Periodontal Management*. The complete treatment procedure was explained to the patient before proceeding with the treatment, a written consent was obtained from him, and a medical consent was obtained from the general physician.

**Figure 3:**
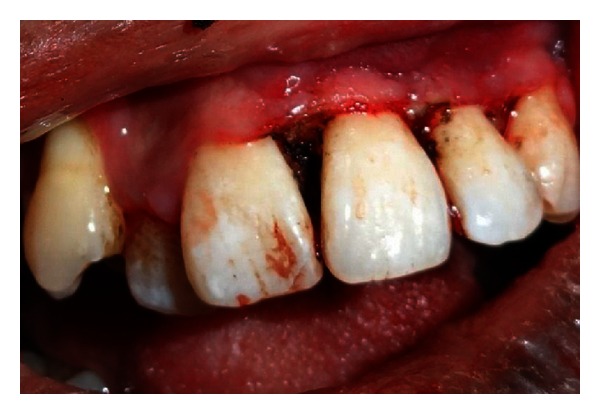
Immediate postoperative photograph.

**Figure 4:**
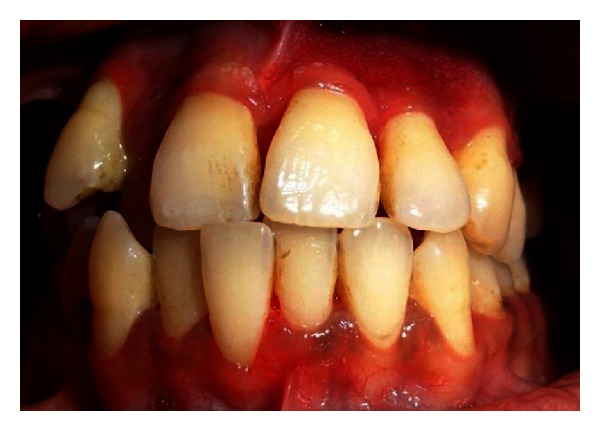
3 months postoperative photograph.

2% lidocaine containing 1 : 80,000 adrenaline infiltration was given on the labial side in the maxillary anterior region and nasopalatine region. The swelling in relation to 11, 21–25 was excised at marginal gingiva, interdental papilla and attached gingiva with diode laser (Picasa 810 nm 1.8 W continuous mode) (Figure 3). The excised tissue was kept in formalin (10%) and sent for histopathologic examination. The patient was advised to use 0.2% chlorhexidine mouth rinse for 2 weeks postoperatively and analgesics as and when required. The patient was asked to return on postoperative day 7. The healing was progressing satisfactorily without any overt complications. At 3 months postoperatively (Figure 4) the healing was uneventful and no recurrence of gingival overgrowth was observed. 

*Case 2*. A 32-year-old male patient was referred to the Department of Periodontics, Sri Sai College of Dental Surgery, with chief complaint of swelling in the left upper region for 2 years which was gradually increasing and associated bleeding from the gums while eating or sometimes spontaneously. He gave no history of convulsions or glaucoma. On further elaborated questioning regarding his medical history he revealed liver cirrhosis caused by hepatitis B. Family history was noncontributory for similar findings in his immediate or distant relatives.

**Figure 5:**
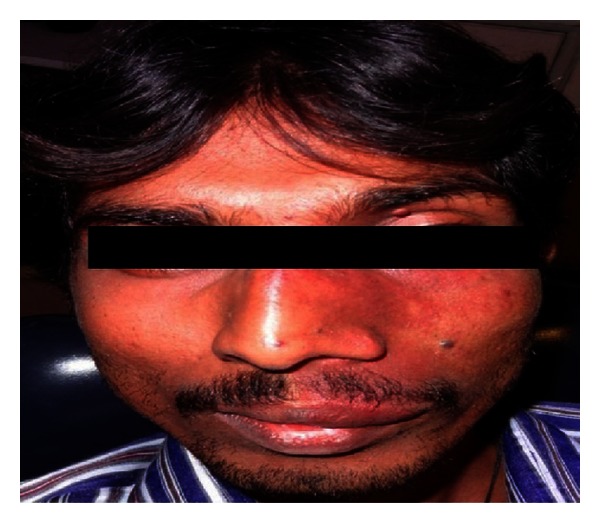
Extraoral view showing port-wine stain on the left side of the face.

**Figure 6:**
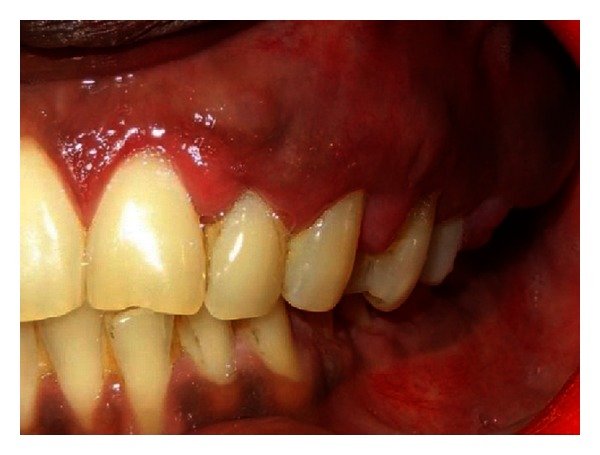
Intraoral view showing gingival overgrowth.

Extraoral examination revealed a unilateral port-wine stain or nevus flammeus along multiple segments of trigeminal nerve on the left of the face (Figure 5). This lesion was present on the face since birth. There was noticeable facial asymmetry on the left side of the face with noticeable swelling of the left side of the upper lip (ipsilateral labial angioma). Intraoral examination (Figure 6) reveals gingival overgrowth which was highly inconsistent with the amount of plaque present. The gingival overgrowth was reddish-pink irt 24–27 involving marginal and interdental papilla of 24-25 with diffuse enlargement in relation to 26-27. The enlargement extended palatally too in relation to 25–27. Bleeding was present on slight provocation. Radiographic, blood investigations and liver function tests were carried out. Detailed clinical examination, history, and investigation confirmed the diagnosis of Sturge-Weber syndrome. 

*Periodontal Management*. Nonsurgical periodontal therapy was done under strict aseptic conditions. The surgical treatment in this case was deferred as the patient was not a candidate for periodontal surgery as the patient is diagnosed with liver cirrhosis. 

*Case 3*. A 12-year-old female patient came to the Department of Periodontics, Sri Sai College of Dental Surgery at Vikarabad, with a chief complaint of swelling in the upper front region for 1 year which was gradually increasing in size with associated gum bleeding while eating or sometimes spontaneously. She gave no history of convulsions or glaucoma. She revealed no relevant family history.

**Figure 7:**
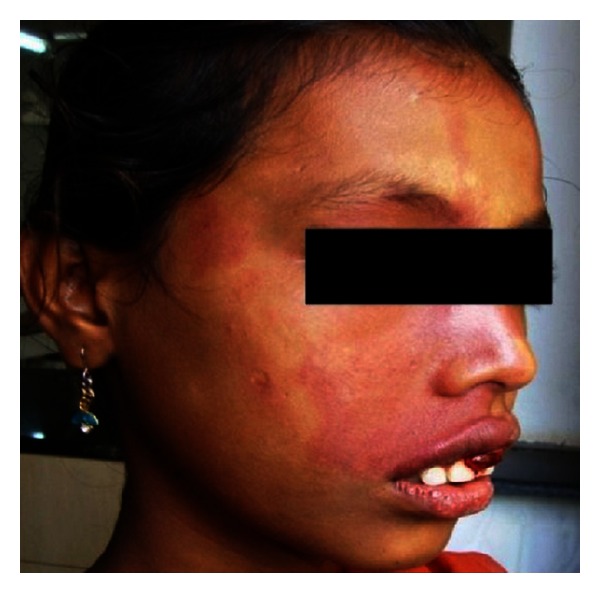
Extraoral view showing port-wine stain on the right side of the face.

**Figure 8:**
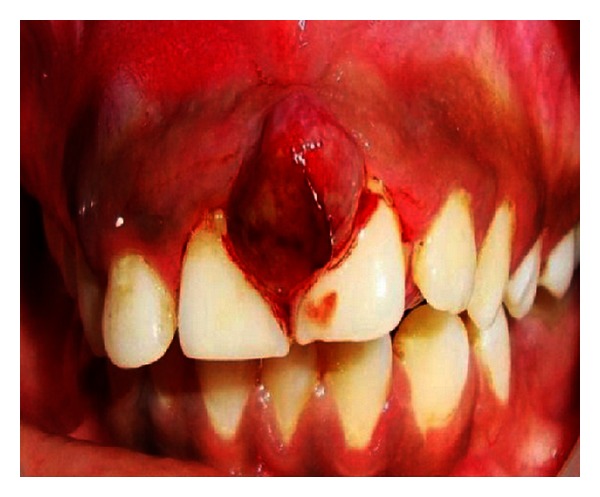
Intraoral view showing unilateral overgrowth.

Extraoral examination revealed the port-wine stain or nevus flammeus along multiple segments of trigeminal nerve on the right side of the face since birth (Figure 7). Reddish discoloration of the sclera of the right eye was seen. Intraoral examination (Figure 8) revealed discrete, sessile, reddish-pink gingival overgrowth in relation to 11 and 21 involving labial and palatal gingiva. Loss of Knife edge contour and bulbous papillae are seen in relation to 11 to 24 both labially and palatally. There was slight blanching of the gingival enlargement on pressure raising concerns that it might be an angiomatous (vascular) nature of gingival enlargement. Bleeding was present on slight provocation. Radiographic and blood investigations were carried out which revealed no abnormality. Histopathological examination (Figure 11) was suggestive of inflammatory fibroepithelial hyperplasia with vascular proliferation. Detailed clinical examination, history, and investigation confirmed the diagnosis of Sturge-Weber syndrome. 

*Periodontal Management*. The complete treatment procedure was explained to the patient and her parents before proceeding with the treatment, a consent form was obtained from her parents, and a medical consent was obtained from the general physician. Cause-related therapy was done before the surgical intervention.

**Figure 9:**
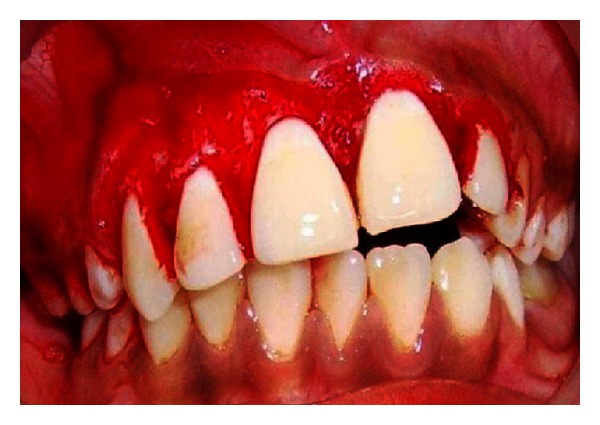
Immediate postoperative photograph.

**Figure 10:**
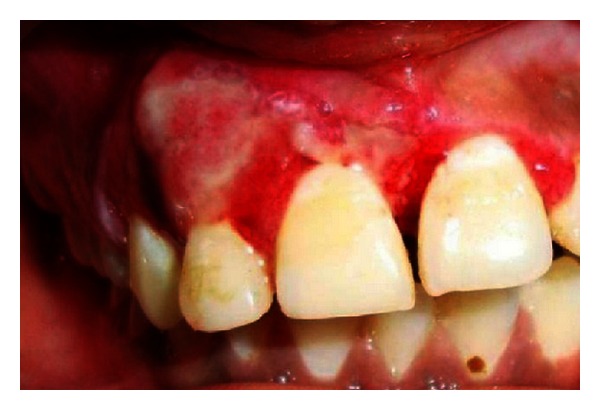
1-week postoperative photograph.

**Figure 11:**
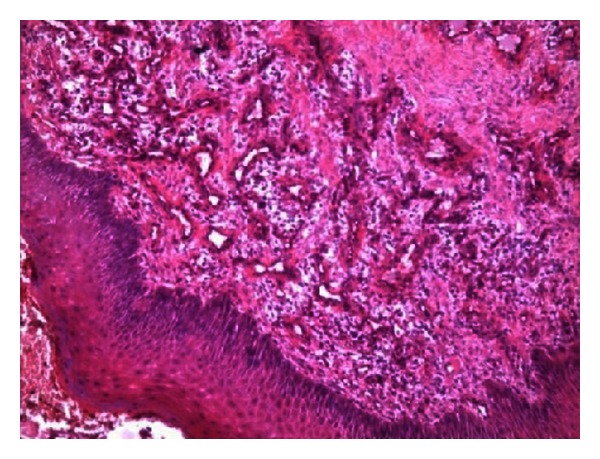
Histopathological view of overgrowth.

2% lidocaine containing 1 : 80,000 adrenaline infiltration was given on the buccal side in the maxillary anterior region and nasopalatine region. The pedunculated swelling irt 11 and 21 was excised initially followed by an external bevel gingivectomy performed irt 12, 13, and 14 with a Bard parker no. 15 surgical blade by excising the marginal gingiva, interdental papilla, and attached gingiva. After the excision (Figure 9) of the gingival enlargement, the bleeding points were cauterized with electrocautery. The bleeding was controlled with pressure packs. The excised tissue was kept in formalin (10%) and sent for histopathologic examination. The patient was kept under close observation for 24 hours for any postoperative complications. The patient was advised to take antibiotic and analgesic regimen (amoxicillin 250 mg, three times a day; Ibugesic kid, two times a day) for 3 days and use 0.2% chlorhexidine mouth rinse for 2 weeks postoperatively. The patient was discharged on postoperative day 2 and was asked to return on postoperative day 7 (Figure 10). The healing was progressing satisfactorily without any overt complications. The patient was asked to return after 15 days for postoperative checkup.

## 3. Discussion

SWS has extremely varied clinical features, and its diagnosis can be characterized by port-wine stain on the face followed by other signs such as glaucoma, epilepsy, and mental retardation.

According to Inan and Marcus [6], the port-wine nevus is localized in the face, especially on the right side of the face, and detected in 87–90% of the cases. The lesion extension over the midline is observed in 50% of the patients and bilateral involvement in 33% of the cases. In case 3, the patient showed nevus flammeus only on the right side without extension over the middle line. Cases 1 and 2 showed nevus flammeus on the left side of the face. Inspite of the absence of neurological disorders, the distribution and extension of the skin angioma were suggestive of SWS in all the three cases. Case 3 was referred to as incomplete SWS and based on Roach scale, this patient was classified Type II SWS as only facial angiomas alone without CNS involvement.

Case 2 was referred to as incomplete SWS and based on Roach scale, this patient was classified Type II SWS as only facial angiomas alone without CNS involvement.

Case 1 was referred to as incomplete SWS and based on Roach scale, this patient was classified Type II SWS. In this case, laser [7] proved advantageous in improving surgical site visibility, bloodless field, and reducing the time of surgery, compared to conventional procedure. No noticeable postsurgical bleeding and discomfort were noted.

The treatment of SWS is variable and depends on the nature or intensity of its clinical features. Usually the port-wine stain may be improved by laser therapy or cosmetics [8], anticonvulsants for epilepsy, and glaucoma treatment to reduce intraocular pressure. One of the most important aspects of the treatment of SWS patients is psychological counselling of patients as well as parents.

## 4. Conclusion

The great frequency of oral manifestations of SWS demands dentists knowledge of this syndrome clinical features and treatment modalities. The consultations from various medical specialists are needed for proper treatment along with professional counselling which benefits the patient and the family to overcome their difficulties and improve the prognosis. Periodic systemic and oral examinations are recommended to prevent any cranial and oral complications. Laser, with its good haemostatic properties, is the best alternative in the surgical treatment of patients at great risk of haemorrhage, such as patients with SWS.
